# Ensemble classification and segmentation for intracranial metastatic tumors on MRI images based on 2D U-nets

**DOI:** 10.1038/s41598-021-99984-5

**Published:** 2021-10-19

**Authors:** Cheng-Chung Li, Meng-Yun Wu, Ying-Chou Sun, Hung-Hsun Chen, Hsiu-Mei Wu, Ssu-Ting Fang, Wen-Yuh Chung, Wan-Yuo Guo, Henry Horng-Shing Lu

**Affiliations:** 1 Institute of Statistics, National Yang Ming Chiao Tung University, Hsinchu, Taiwan; 2 Department of Radiology, Taipei Veterans General Hospital, Taipei, Taiwan; 3 Center of Teaching and Learning Development, National Yang Ming Chiao Tung University, Hsinchu, Taiwan; 4 Department of Neurosurgery, Kaohsiung Veterans General Hospital, Kaohsiung, Taiwan

**Keywords:** Computational science, Cancer imaging

## Abstract

The extraction of brain tumor tissues in 3D Brain Magnetic Resonance Imaging (MRI) plays an important role in diagnosis before the gamma knife radiosurgery (GKRS). In this article, the post-contrast T1 whole-brain MRI images had been collected by Taipei Veterans General Hospital (TVGH) and stored in DICOM format (dated from 1999 to 2018). The proposed method starts with the active contour model to get the region of interest (ROI) automatically and enhance the image contrast. The segmentation models are trained by MRI images with tumors to avoid imbalanced data problem under model construction. In order to achieve this objective, a two-step ensemble approach is used to establish such diagnosis, first, classify whether there is any tumor in the image, and second, segment the intracranial metastatic tumors by ensemble neural networks based on 2D U-Net architecture. The ensemble for classification and segmentation simultaneously also improves segmentation accuracy. The result of classification achieves a F1-measure of $75.64\%$, while the result of segmentation achieves an IoU of $84.83\%$ and a DICE score of $86.21\%$. Significantly reduce the time for manual labeling from 30 min to 18 s per patient.

## Introduction

Cancer is a disease that occurs when malignant cells grow in the body. These cells can form almost anywhere, including the brain, lungs, pancreas, and more. Cancerous cells cluster together to form a mass called a tumor and can spread throughout the body to other, more distant areas. In this article, we focus on the brain metastases which occur when cancer cells spread from their original site to the brain. Furthermore, an estimated 8–10% of adults with symptomatic brain metastases^1^, which is one of the most serious consequences, and it is essential to determine its stage. After diagnosis, it is necessary to take a treatment from the initial stage of cancer, such as gamma knife radiosurgery (GKRS) which is the major therapeutic strategy in this article.

GKRS is a type of radiation therapy used to treat tumors, vascular malformations, and other abnormalities in the brain. During GKRS, 201 cobalt 60 sources are arranged in a hemispheric shape in such a manner that all the gamma rays are focused at the center to create a cumulative radiation field^2^. The total energy of the rays is therefore transmitted precisely to the target with the preservation of normal brain tissue so that the lesion will degenerate^2^. To have this operation before, the radiologist should confirm the coordinate of the lesion on the MRI images. This treatment provides a new method for traditional neurosurgery and makes the surgical procedure be safer and faster. However, the time it takes for the radiologist to mark the location of the lesion on the MRI images depends on the shape and the volume of the tumors, and it often takes more than 30 min on average. It will even take several times longer to confirm the result in detail again.

This article developed an automated deep learning assistant diagnosis model for radiography. It can shorten the time to interpret the location of the lesion; moreover, it serves as a second suggestion for the doctors. Especially, for patients with terminal cancer, after the patient undergoes an MRI examination, the radiologist can quickly make a diagnosis report based on the preliminary data of the auxiliary diagnosis. Doctors can diagnose brain metastases, determine the stage of cancer, and develop treatment policies as soon as possible, which can greatly reduce the waiting time of patients. Relatively, doctors can also provide patients more care and design the best treatment strategies to make patients less anxious. In addition, it may speed up the hospital process, save hospital operating costs, and enhance competitiveness.

Our solution is to perform 2D U-Nets in ensemble learning for intracranial metastatic tumors classification and segmentation. The dataset contains the post-contrast $T_{1}$-weighted whole-brain MRI images which are an important clinical tool for cancer detection, diagnosis, prognosis, and treatment evaluation. In brief, the dataset is split as train, validation and test set first. We use train set to build a 2D U-Net model, and input validation set to this model to select an image classification threshold as the Youden index given by Receiver Operating Characteristic (ROC) curve. Furthermore, for segmentation, we calculate the detection rate of tumors in Precision-Recall (PR) curve, then set the mask threshold by the maximal F1-measure for labelling if a pixel has tumor. Finally, the test set is input to the model and we use the thresholds obtained above for classification and segmentation. In the last, the Intersection over Union (IoU) and DICE score are used as metrics for evaluation.

The contribution of this article is to design an automated 2D intracranial metastasis tumor segmentation system to overcome common problems in practice, such as hardware equipment, insufficient data and imbalanced data. Overview deep learning papers or related competitions on medical images of the brain, nothing more than using 3-dimensional models for construction or excellent hardware equipment to train a good model to refresh the ranking of the competition and publish a paper. However, in practice, there is no such abundant resource that can make AI produce the best possible results. Therefore, this article puts forward a new idea based on this position. Further design the ensemble strategy for classification and segmentation simultaneously, reducing the time for manual labeling from 30 min to 18 s per patient.

## Materials and methods

### Materials

**Figure 1:**
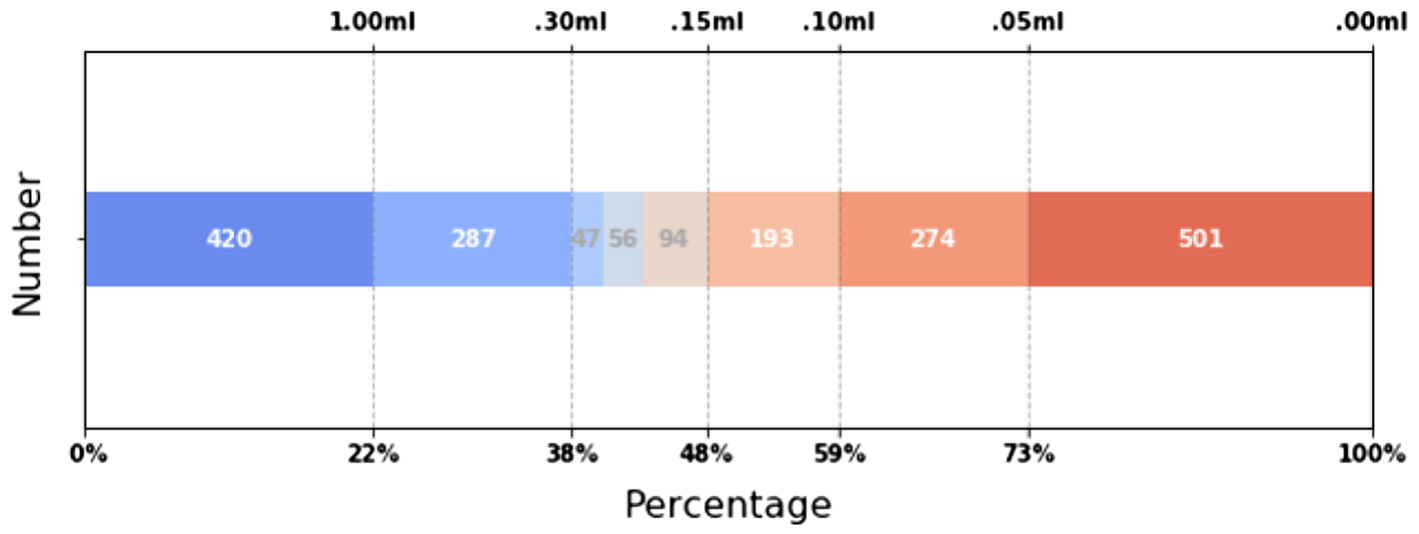
The number and proportion of each tumor volume range.

**Figure 2:**
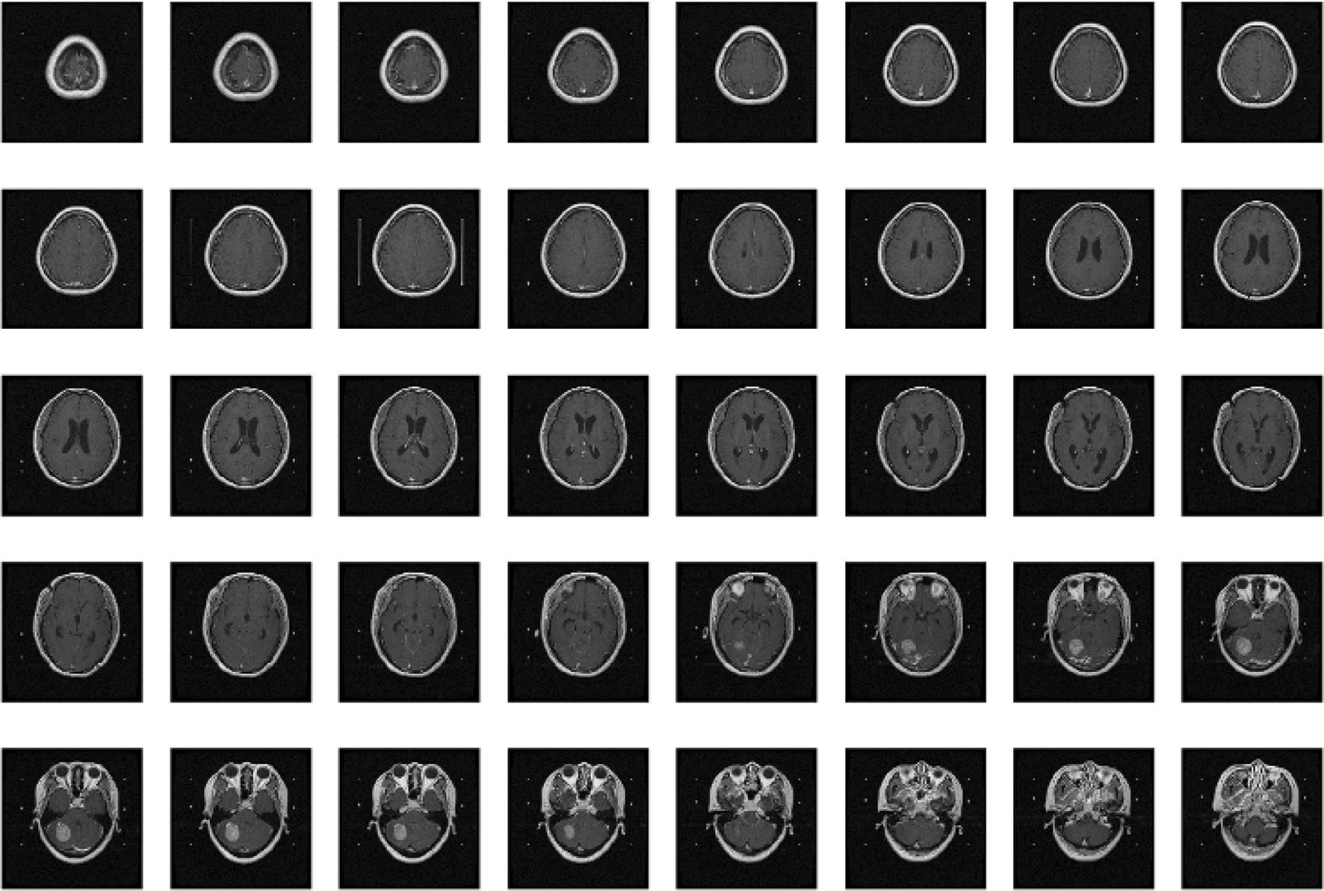
Post-contrast T1-weighted MRI per patient.

The dataset of MRIs were originally acquired for guiding radiosurgical treatment using the Gamma Knife from 1999 to 2018. This dataset contained 556 patients suffered from intracranial metastases and referred for GKRS, including the precise lesion locations of total 1872 metastases lesions had been collected. This retrospective study was approved, and informed consent was waived by the Institutional Review Board of Taipei Veterans General Hospital (IRB-TPEVGH No.: 2017-10-017AC) and conformed to the tenets of the Declaration of Helsinki. All methods were carried out in accordance with relevant guidelines from IRB-TPEVGH. This article investigated tumors with a volume greater than 0.15 ml. In Fig. 1, the reason is that the size of the tumor accounts for almost half of the total number of tumors. A total of 492 patients with 904 tumors and 23354 $T_{1}$-weighted MRI images were found that an average of 48 images per patient, $20\%$ of which had tumors, showed an imbalanced dataset. The image format is stored with the size of $512\times 512$ pixels (approximately 5 mm per pixel) and the thickness of 3 mm. The original image of the whole brain in per patient will be presented in Fig. 2. In order to generate a corresponding mask for each image to use in the calculation of tumor volume, several DICOM tags need to be used.

#### Data pre-processing

The pre-processing step in this study involves three parts, selection for the region of interest (ROI), enhancement, and normalization. The pre-processing step firstly selects the ROI by the active contour model to overcome the imbalance pixel number for the skull and the background. Then, we enhance the contract to strengthen the image information. We also resize the image because the image size of ROI is varying. To reduce the noise in the MRI images, the snake model was utilized for delineating the skull outline, which makes use of the energy constraints and forces in the image for separation of the region of interest (ROI)^3,4^. The reason for this action is to overcome the imbalance pixel number for the skull and the background. Only focus on address the ROI part of image enhancement by z-score; besides, set the background to all black for decreasing the subsequent impact on convolutional neural network (CNN) training. Considering the structure of the models and the scale of the dataset, resize the image to $256\times 256$ and normalize the data dimensions so that each image is approximately the same scale which the minimal and maximal along the dimension is $-1$ and 1^5^. At last, fill the background with intensity value zero again to ensure the image does not appear other values.

#### Methods

**Figure 3:**
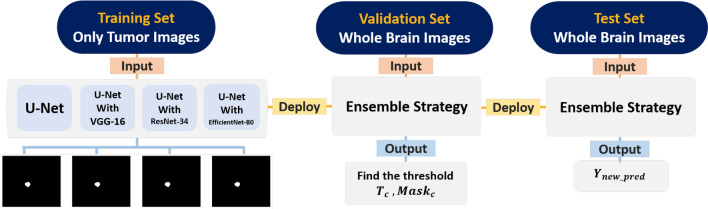
The framework of the experimental design in this article.

Figure 3 provides the framework of the experimental design. Input the processed images into four semantic segmentation models individually, 2D-UNet, 2D-UNet with backbone VGG-16, 2D-UNet with backbone ResNet-34, and 2D-UNet with backbone EfficientNet-B0, then get four different outputs, namely the metastasis probability maps^6–9^. For the evaluation, deploy the trained model to the validation set for determining the threshold of classification and segmentation. Input the test set through the function $I_{T_{c}}(score_{i})$ which do the classification is given by Eq (1)

1 $$\begin{aligned} Y_{pred} = I_{T_{c}}(score_{c,i}) = {\left\{ \begin{array}{ll} \text {All Black} &{} \text {if}~{ score_{i} < T_{c}}\\ Mean(f_{1,i}, f_{2,i}, f_{3,i}, f_{4,i}) &{} \text {otherwise} \end{array}\right. } \end{aligned}$$

where

$$\begin{aligned} \begin{array}{llll} score_{i} &{} \text {Median}(\max (f_{1,i}), \max (f_{2,i}), \max (f_{3,i}), \max (f_{4,i})) \\ f_{j,i} &{} \text {the} i^{th} \text {probability maps of the} j^{th} \text {model} \\ T_{c} &{} \text {Youden Index} \\ \end{array} \end{aligned}$$

In classification, select the image threshold $T_{c}$ by ROC curves to determine whether the image had tumors or not. If the predicted image had tumors, do the semantic segmentation by average the images $f_{j,i}$ from four different models. Collect all the result be the $Y_{pred}$ in this part, namely stage-one.

Next, the mask threshold level is selected where all pixel values below the threshold are mapped to zero (black) and an upper threshold value is chosen so that all pixel values above this threshold are mapped to one (white). Then the binary masks $Y_{new\_pred}$ for an predicted image would be created. In Eq. (2), if the *g*(*x*, *y*) were the input image, the thresholded image *h*(*x*, *y*) is given by

2 $$\begin{aligned} h(x,y) = {\left\{ \begin{array}{ll} 1 &{} \text {if}~{ g(x,y) > Mask_{c}}\\ 0 &{} \text {otherwise} \end{array}\right. } \end{aligned}$$

Finally, computed the performance of the new output prediction $Y_{new\_pred}$.

#### Experimental environment

The network has been implemented using the open source Keras framework on top of TensorFlow^10^. For the training process, each 2D U-Net model requires approximately 2 hours to finish on an NVIDIA GeForce GTX 1080 Ti graphics processing unit (GPU) with 11G memory and Intel®Core(TM) i7-6700 CPU @ 3.40GHz with 64GB random access memory (RAM).

## Results

Evaluating the framework on the validation set with 80 patients and the test set with 100 patients both using the whole brain images. The performance of classification was evaluated by ROC curve, AUC, precision, and Recall^11,12^. The segmentation part was measured by PR curve, F1-measure, IoU and DICE^13^.

### Classification

**Figure 4:**
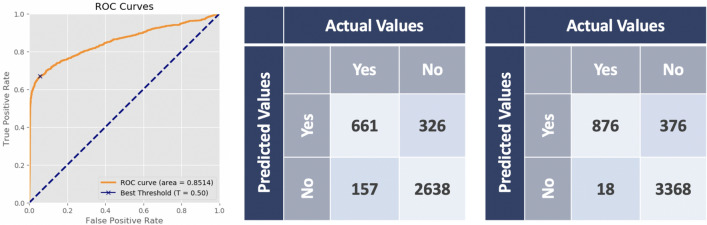
The result in classification. The left is the ROC curve of the classification. At $T_{c}=0.5$, middle shows the confusion matrix from validation set, the right is from the test set.

Use validation set to determine decision points and plot the ROC curve of the result in classification. As shown in Fig. 4, the AUC were 0.8514 in validation set, which around 0.8 to 0.9 is considered excellent^4^. According to the Youden Index, the best threshold $T_{c}$ was 0.50. The confusion matrix of the classification in Fig. 4 indicated that the precision and Recall in Table 1.

In test set, given the $T_{c}=0.5$, if the $score_{i}$ calculated in stage-one was less than 0.50, then the image will be present in no tumors. Figure 4 presented the confusion matrix of the classification in test set. The Youden Index ensured the number of false negative be the minimal in this set, so the images were not missed. In addition, the precision and recall were shown in Table  1.

**Table 1:** Classification performance.

	Validation set	Test set
*Precision*	66.97%	69.96% (0.6600, 0.7380)
*Recall*	73.24%	82.33% (0.7894, 0.8544)
	Validation set	Test set

#### Segmentation

**Figure 5:**
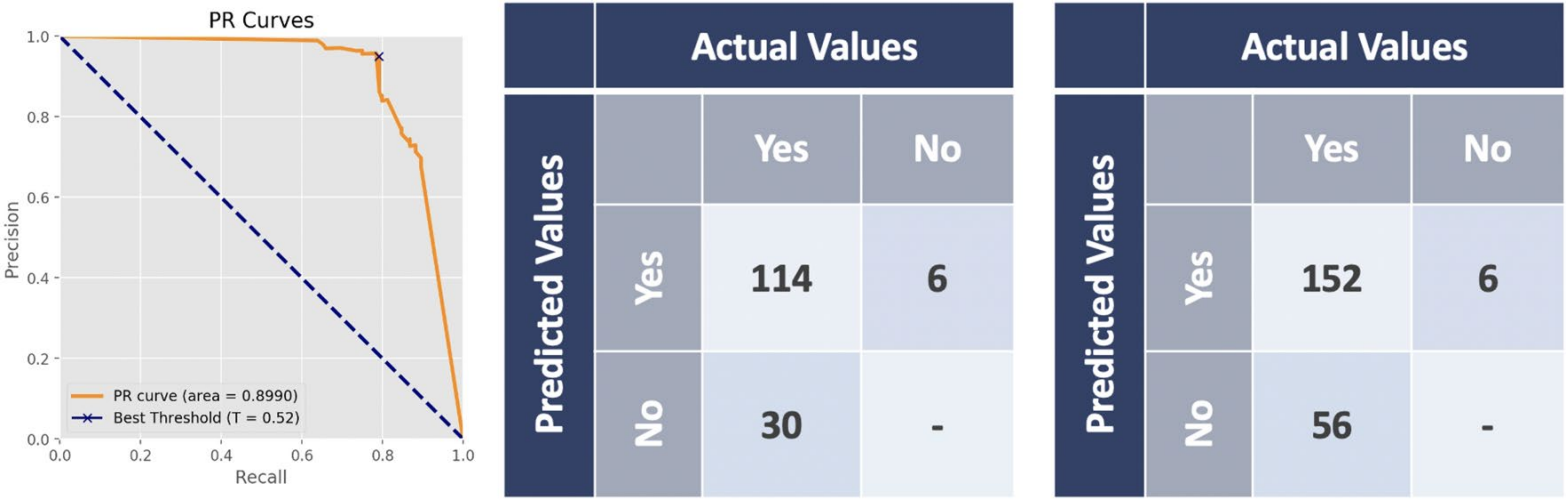
The result in segmentation. The left is the PR curve of the detection rate of tumors. At the $Mask_{c}=0.52$, middle shows the confusion matrix from validation set, the right is from the test set.

Undertake the images classified as having tumors, then averaged the image segmentation results in validation set for determined the $Mask_c$. Through different mask thresholds, the PR curve is drawn to obtain the AUC of the tumor volume bigger than 0.15 ml detected rate of the validation set was 89.90%, in which the value around 0.8–0.9 is extremely good; that is, a certain level of the tumor can be detected through this experimental. Figure 5 and Table 2 shown the performances in $Mask_{c}=0.52$, which the maximal of F1-measure on the PR curve. Next, calculated the IoU and DICE score of each image which the performances shown in Table 2.

In test set, given the best mask threshold $Mask_{c}=0.52$ from the validation set, which means that all intensity values below the threshold are mapped to zero (black) and an upper threshold value is chosen so that all intensity values above this threshold are mapped to one (white). So the binary masks were be the final prediction results of ensemble strategy. Then, calculate the IoU and DICE score of each image in the test set, and the measurement to get the results in Fig. 5 and Table 2.

**Table 2:** Segmentation performance.

	Validation set	Test set
*Precision*	95.00%	96.20% (0.9314, 0.9876)
*Recall*	79.17%	73.08% (0.6568, 0.8077)
$F1-measure$	86.36%	83.06% (0.7704, 0.8886)
*IoU*	84.43%	84.83% (0.8259, 0.8665)
*DICE*	85.68%	86.21%(0.8413, 0.8790)

#### Visualization

**Figure 6:**
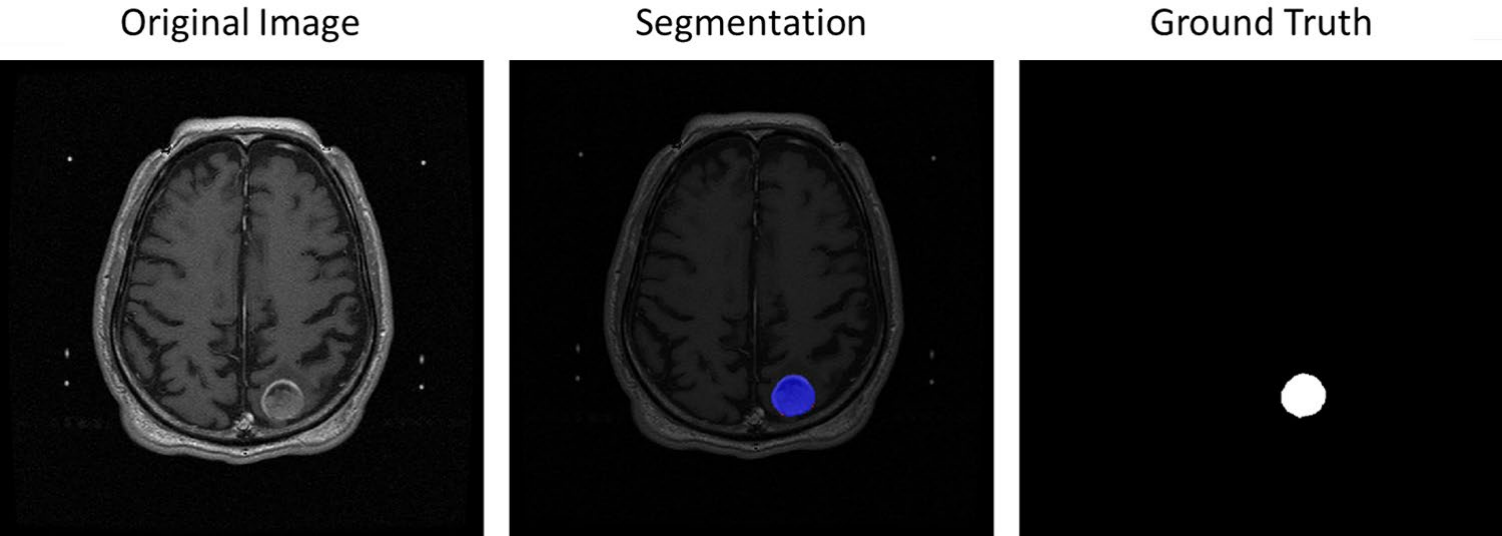
Selected the tumor segmentation results compared in slice-by-slice.

**Figure 7:**
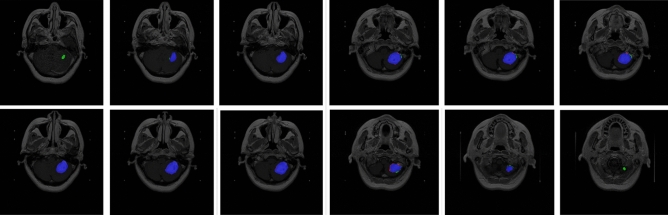
Visualization the segmentation by the ensemble strategy.

Figure 6 visualize the segmentation results. The left-hand side was the original MRI image and the right-hand side shown the ground truth where the tumors was in the slice. The middle is the segmentation, the blue part was the true positive, the green region was the false negative and the area in red was false positive. More segmentation result shown in Figure 7.

## Conclusion and comparison

The main contribution of this study is to design an automated 2D intracranial metastasis tumor segmentation system in practice. Significantly shorten the time for radiologists to mark lesions. Through a fully automated process, the MRI of a patient can be completed about every 18 s. In this way, doctors can also spend more time taking care of the psychological state of patients and their families; therefore, improve the comprehensive medical quality.

The technical part in this study, image pre-processing and two-stage ensemble strategy consider to overcome the imbalanced classification and the pixel-wise class imbalanced in binary image segmentation. Make good use of the characteristics of the semantic segmentation model and use different types of datasets, design a ensemble strategy for classification and segmentation simultaneously. Moreover, ensemble modelling improves segmentation accuracy. The performance of the model also exceeds the 3D U-Net.

This study proposes the integrated and automatic approach to classify and segment the intracranial metastatic tumors from MRI images. The proposed approach firstly classifies whether there is any tumor in the image. If this is positive, then it segments the intracranial metastatic tumors by ensemble learning based on the 2D U-Net architecture. For the first part of classification, recent studies^14–16^ have similar results. In^14^, they use the 3D architecture in CNN to classify seven classes for health and various tumor types. They also conduct the comparison for quantitative results by 2D ResNet 50. In the comparison, the sensitivity, which is equal to Recall in our paper, are 72, 72, 92, 94, 97, 83 and 57 in percentage, whereas our result is 82.33 in percentage. Note that, their sample sizes range from 45 to 359, whereas our sample size is 23,354. In^15,16^, they use the method of mRMR as the feature selection method and the similar architecture to classify the images. In^15^, they focus on the breast and use ultrasonography as input images. In^16^, they proposed the hybrid models with KNN to achieve great performance in F1-score and sensitivity. However, their total samples are 12,800 for four classes and they do not develop the segmentation part. Hence, the integrated and automatic approach developed in this study is more suitable for clinical practice because it provides comprehensive tools for medical experts.
